# Calcified Hyaline Degeneration Nodule in the Upper Lip Mucosa: A Rare Case Report

**DOI:** 10.1155/crid/5553158

**Published:** 2025-12-12

**Authors:** Changjun Huang, Yujun Lu, Kui Huang, Fang Chen, Yajing Wang, Yingkun Luo

**Affiliations:** ^1^ The Affiliated Stomatological Hospital of Guizhou Medical University, Guizhou Medical University, Guiyang, Guizhou Province, China, gmc.edu.cn; ^2^ Department of Stomatology, People′s Hospital of Dafang, Bijie, Guizhou Province, China

**Keywords:** calcification, hyaline degeneration, oral mucosal nodule

## Abstract

Hyaline degeneration is a common degenerative disease after tissue or cell injury. It can occur in diseases such as renal fibrosis, atherosclerosis, and oral submucous fibrosis. Homogeneous eosinophilic deposits characterize it. However, the occurrence of hyaline degeneration is rarely accompanied by calcification. We present a rare case of a 33‐year‐old female patient diagnosed with a calcified nodule of the upper lip mucosa, which was confirmed as a benign entity without malignancy. This report is aimed at enhancing understanding of this condition and providing a reference for future diagnosis and treatment strategies.

## 1. Introduction

Hyaline degeneration is a common degenerative disease after tissue or cell injury, characterized by homogeneous eosinophilic deposits under HE staining. Histologically, it presents as extensive fusion of collagen fibers accompanied by hyaline degeneration. For example, kidney damage will be accompanied by the formation of renal fibrosis. At the end of renal fibrosis, most glomeruli will show extensive fibrosis and hyaline degeneration pathological changes [1]. Amyloidosis has clinical manifestations similar to those of hyaline degeneration, but histologically, the formation of fiber bundles is primarily due to amyloid protein deposition, without evidence of fiber fusion or hyaline degeneration [2]. However, the occurrence of hyaline degeneration is rarely accompanied by calcification [3]. Given the rarity of this presentation, we report a case of a calcified hyaline degeneration nodule in the upper lip mucosa, aiming to contribute to the diagnostic literature and support future related research.

## 2. Case Presentation

A 33‐year‐old female patient was admitted to the Department of Stomatology, People′s Hospital of Dafang, on July 10, 2024, due to a painless mass on her upper lip. The patient noticed this mass more than 2 years ago; she reported no pain, no noticeable growth or regression, and had not received any treatment. The patient had no systemic diseases such as hypertension, diabetes, heart disease, no infectious diseases such as hepatitis B, tuberculosis, AIDS, no long‐term medication history, no trauma history, no surgery history and blood transfusion history, no drug allergy history, and no family genetic history.

**Figure 1:**
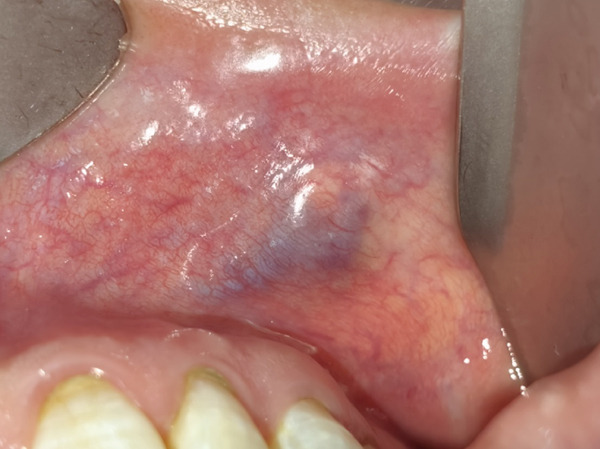
Clinical photos from initial presentation. The tumor was located under the lip mucosa; the surface was light blue, nodular hyperplasia.

The patient′s maxillofacial structure was essentially symmetrical. Her mouth opening was normal, with no tenderness in the bilateral temporomandibular joint area, and her condylar movement was consistent. A round mass, approximately 0.5 × 0.5 cm in size, was palpable on the medial side of the left upper lip. It was nodular, firm, and bone‐like, with well‐defined borders, good mobility, and visible submucosal vascular proliferation (Figure 1).

The upper lip nodule was surgically removed (Figure 2a). The nodule was yellow–white (Figure 2b), and the section showed a solid gray–white appearance. After HE staining, homogeneous, structureless pink‐stained areas can be observed at the margin of the nodule. A large amount of calcification (dark blue–stained) was observed in the internal area of the nodule. At the peripheral tissue of the nodule, there was a large amount of chronic inflammatory cell infiltration (Figure 3). Six months after the operation, the incision had healed well with no signs of recurrence (Figure 2c). The summary clinical progression timeline of this patient is shown in Table 1.

Figure 2Clinical photos from surgery. (a) The nodule was grayish white; (b) intraoperative isolated specimens; (c) 6 months postsurgery.

**Figure:**
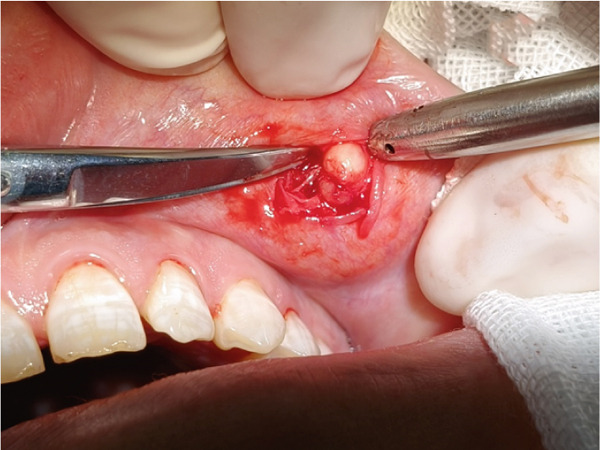
(a)

**Figure:**
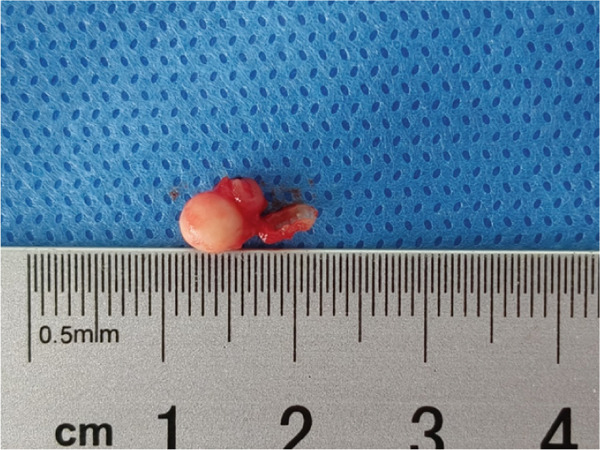
(b)

**Figure:**
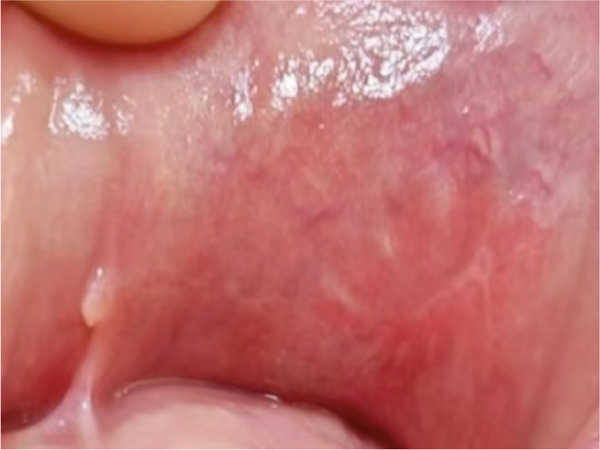
(c)

Figure 3Pathological and immunohistochemical images. Histopathological sections showed benign hyperplasia, fibrous tissue hyperplasia, and hyaline degeneration in the tubercle margin, calcification in the internal area of the nodules, and chronic inflammatory cell infiltration in some areas. (a) ×40; (b) ×200.

**Figure:**
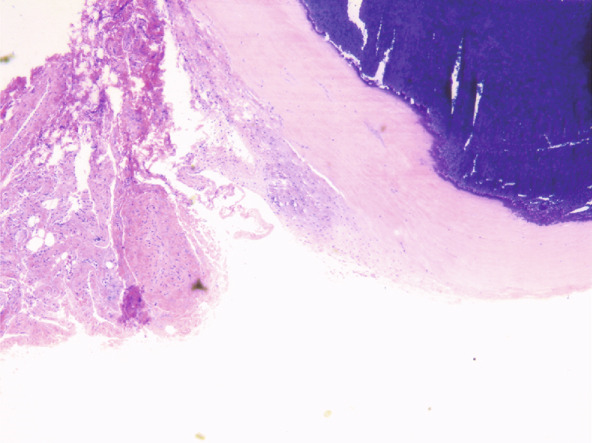
(a)

**Figure:**
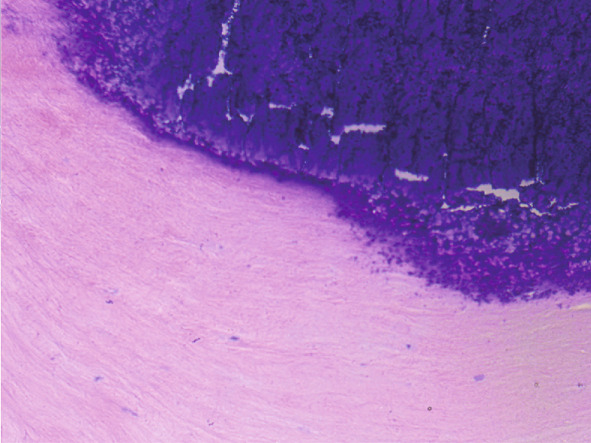
(b)

**Table 1:** The clinical progression timeline of this patient with calcified hyaline degeneration nodule in the upper lip mucosa.

**Date**	**Clinical progression**
July 10, 2024 (first visit)	This patient underwent supragingival scaling and a complete blood count examination.
July 11, 2024	After excluding surgical contraindications and signing an informed consent form, an excisional biopsy of the nodule was performed under local anesthesia.
July 18, 2024	The histological exam released.
July 19, 2024	The patient showed good wound healing. The sutures were removed.
February 20, 2025	The patient showed no discomfort, and the nodules had not recurred.

## 3. Discussion

The clinical features of the patient were round, hard nodules with good mobility, vascular proliferation and varicose veins on the surface. Based on the patient′s clinical manifestations, our department initially diagnosed the condition as left upper lip fibroma. This diagnosis was supported by its alignment with the clinical characteristics of fibroma, including an exophytic, firm, well‐defined, and asymptomatic nodule with a pink or flesh‐colored appearance [4]. Neurofibromas and schwannomas are often differentiated from fibromas. However, biopsy after surgical resection revealed that the pathological features were primarily hyaline degeneration and calcification. In contrast, the pathological features of fibroma resemble inflammatory hyperplasia of the tissue, mainly presenting as epithelial hyperplasia and hyperkeratosis. In the connective tissue, dense collagen fibers and focal hyperplasia of mature fibroblasts were observed, with minimal or no inflammatory cell infiltration [5]. In schwannomas, palisading nuclei and Verocay bodies can be found [6]. Spindle‐shaped cell nuclei can be seen in neurofibromas, and mast cells are occasionally present [7]. Therefore, this case cannot be diagnosed as a fibroma, neurofibroma, or schwannoma.

Hyaline degeneration is a common degenerative disease after tissue or cell injury. The accumulation of homogeneous, translucent, and eosinophilic vitreous material characterizes it. It is clinically observed to be gray and white lesions by the naked eye. This hyalinized material replaces the normal tissue structure, resulting in decreased elasticity, hardening, and impaired function. For example, atherosclerosis, as a chronic cardiovascular disease, has pathological features that are often accompanied by hyaline degeneration, which leads to vascular wall sclerosis and cardiovascular dysfunction [8]. Its occurrence is related to inflammatory cells and is positively correlated with the degree of inflammation [9]. Therefore, hyalinosis also often suggests the occurrence of inflammation. Oral hyalinosis diseases that meet the above characteristics are common in oral submucous fibrosis (OSF) lesions. The pathological features are mainly submucous hyalinosis bands of collagen fibers, accompanied by edema between collagen fibers and lymphocyte infiltration. In the late stage, all collagen fibers may experience hyalinosis. This disease mostly occurs among people who chew betel nuts. The clinical features are that the fibrous cord‐like structure can be touched, and the tissue becomes hard, which can easily lead to limited mouth opening and gray changes [10]. However, the nodule did not meet the above diagnostic criteria and therefore could not be diagnosed as OSF.

By excluding the above diagnosis, we speculate that it may be hyaline degeneration caused by calcification, and there are few reports on the causes of calcification in the oral mucosa. In 1992, El‐Mofty and Santa Cruz introduced the term “oral mucosal calcified nodules” to more specifically describe calcifications in the oral submucosa [11]. They pointed out that the formation of calcified nodules is related to trauma or metabolic disorders of calcium and phosphate. Its pathological features are mainly the proliferation of stratified squamous epithelium, the increase of amorphous calcified deposits, and the infiltration of multinucleated giant cells and other inflammatory cells, making the lesions granulomatous. Current research has found that tissue damage leads to the release of phosphate‐binding proteins by dying cells. These proteins bind to phosphorus, leading to calcification, and the elevated levels of calcium and phosphorus further promote crystal formation and cell necrosis. The resulting tumor necrosis factor, IL‐6, and IL‐1*β* accelerate the formation of calcium salts, thereby triggering chronic inflammation and hypoxia in blood vessels. This type of calcification is called dystrophic calcification and is commonly observed in systemic sclerosis, dermatomyositis, and systemic lupus erythematosus [12].

Nutritional dystrophic calcification is often distinguished from phleboliths. Phleboliths can be observed in patients with vascular malformations, where the cause is slowed peripheral blood flow, leading to thrombus formation that subsequently calcifies. The surrounding fibrous tissue is affected, causing secondary calcification and attachment. Its pathological feature is a papillary structure with a fibrous core and an endothelial lining [13]. The stimulating factors for both are different, and their pathological manifestations also differ. Based on this patient′s medical history and clinical examination results, phleboliths cannot be diagnosed.

Therefore, the formation of calcification in the oral cavity may be related to hyaline degeneration. In this case, the massive lesion with hyaline degeneration was in the oral subepithelial connective tissue. Hyalinosis of fibrous connective tissue is gray and white to the naked eye. It is more common in atrophied uterus and breast interstitium [14], scar tissue, atherosclerotic plate, and various necrosis tissues. We summarize the relevant differential diagnoses in Table 2.

**Table 2:** Differential diagnosis associated with this case.

**Disease**	**Pathological features**	**Cause**
Atherosclerosis [15]	Formation of calcified plaques and fibrous caps, accompanied by macrophage infiltration	Thrombotic lesion
Scar tissue [16]	Fibroblasts secrete, tissue fibrosis, no obvious calcification	During the wound healing
Fibroma	Visible are dense collagen fibers and focal hyperplasia of mature fibroblasts, with minimal or no infiltration of inflammatory cells and no obvious calcification	Injury
OSF	Organ fibrosis, with hyaline degeneration visible in the late stage and no obvious calcification	Chewing betel nut

This patient is without systemic disease, which indicates this nodule is not a manifestation of systemic disease in the mouth. In the center of this hyaline degeneration nodule, there was extensive calcification; the occurrence and mechanism of which are not clear. The interplay between abnormal protein metabolism, cellular stress responses, chronic injury, and various other factors may cause the underlying mechanisms.

## 4. Conclusion

Considering the above factors, this case cannot be diagnosed with a specific oral disease. However, since the patient did not experience a relapse after 6 months of follow‐up and the pathological examination showed no malignant changes, the nodule can be confirmed as a benign lesion. At present, the primary treatment is surgical resection. This is a rare case, and no similar cases have been reported. Asymptomatic oral nodules of unknown etiology warrant biopsy to prevent misdiagnosis of rare or atypical pathologies. Future cases should include additional diagnostic methods to investigate the etiology and mechanisms further, providing a basis for accurate diagnosis and targeted treatment.

## Ethics Statement

The photographs used in this case report do not show the identity of the patient. This case report is not a research project; therefore, it does not have an IRB approval number. All the treatment stages were in accordance with the Declaration of Helsinki (1965) and its later amendments. This report was written according to the CARE guidelines.

## Consent

The authors confirm that written informed consent was obtained from the patient for publication of all clinical data and pathological specimens associated with this case. All personally identifiable information has been anonymized through the removal of metadata and alteration of nonessential demographic features.

## Conflicts of Interest

The authors declare no conflicts of interest.

## Author Contributions

Changjun Huang (first author): conceptualization, investigation, resources, data curation, supervision, writing—original draft, writing—review and editing. Yujun Lu: data curation, supervision, writing—review and editing. Yajing Wang and Yingkun Luo (corresponding author): conceptualization, investigation, resources, data curation, supervision, funding acquisition, writing—review and editing. Kui Huang: data curation, supervision, writing—review and editing. Fang Chen: data curation, supervision, writing—review and editing. Yajing Wang and Yingkun Luo contributed equally to this work and share last authorship.

## Funding

This work was supported by the National Natural Science Foundation of China (82260200).

## Data Availability

Data sharing is not applicable to this article as no datasets were generated or analyzed during the current study.
